# Psychometric validation and refinement of the Interoception Sensory Questionnaire (ISQ) in adolescents and adults on the autism spectrum

**DOI:** 10.1186/s13229-021-00440-y

**Published:** 2021-06-07

**Authors:** Evan Suzman, Zachary J. Williams, Jacob I. Feldman, Michelle Failla, Carissa J. Cascio, Mark T. Wallace, Maria Niarchou, James S. Sutcliffe, Ericka Wodka, Tiffany G. Woynaroski

**Affiliations:** 1 Graduate Program in Biomedical Sciences, Vanderbilt University, 1215 21st Avenue South, Medical Center East, Room 8310, Nashville, TN 37232 USA; 2 Department of Hearing and Speech Sciences, Vanderbilt University Medical Center, 1215 21st Avenue South, Medical Center East, Room 8310, Nashville, TN 37232 USA; 3 Vanderbilt Brain Institute, Vanderbilt University, Nashville, TN USA; 4 Frist Center for Autism and Innovation, Vanderbilt University, Nashville, TN USA; 5 Medical Scientist Training Program, Vanderbilt University School of Medicine, Nashville, TN USA; 6 Center for Healthy Aging, Self Management and Complex Care, College of Nursing, The Ohio State University, Columbus, OH USA; 7 Vanderbilt Kennedy Center, Vanderbilt University Medical Center, Nashville, TN USA; 8 Department of Psychiatry and Behavioral Sciences, Vanderbilt University Medical Center, Nashville, TN USA; 9 Department of Psychology, Vanderbilt University, Nashville, TN USA; 10 Vanderbilt Genetics Institute, Vanderbilt University Medical Center, Nashville, TN USA; 11 Department of Molecular Physiology and Biophysics, Vanderbilt University, Nashville, TN USA; 12 Department of Psychiatry and Behavioral Sciences, Johns Hopkins University School of Medicine, Baltimore, MD USA; 13 Center for Autism and Related Disorders, Kennedy Krieger Institute, Baltimore, MD USA

**Keywords:** Autism, Interoception, Psychometric, Reliability, Validity, Sensory, Item response theory, Measurement

## Abstract

**Background:**

Individuals on the autism spectrum are reported to display alterations in interoception, the sense of the internal state of the body. The Interoception Sensory Questionnaire (ISQ) is a 20-item self-report measure of interoception specifically intended to measure this construct in autistic people. The psychometrics of the ISQ, however, have not previously been evaluated in a large sample of autistic individuals.

**Methods:**

Using confirmatory factor analysis, we evaluated the latent structure of the ISQ in a large online sample of adults on the autism spectrum and found that the unidimensional model fit the data poorly. Using misspecification analysis to identify areas of local misfit and item response theory to investigate the appropriateness of the seven-point response scale, we removed redundant items and collapsed the response options to put forth a novel eight-item, five-response choice ISQ.

**Results:**

The revised, five-response choice ISQ (ISQ-8) showed much improved fit while maintaining high internal reliability. Differential item functioning (DIF) analyses indicated that the items of the ISQ-8 were answered in comparable ways by autistic adolescents and adults and across multiple other sociodemographic groups.

**Limitations:**

Our results were limited by the fact that we did not collect data for typically developing controls, preventing the analysis of DIF by diagnostic status. Additionally, while this study proposes a new 5-response scale for the ISQ-8, our data were not collected using this method; thus, the psychometric properties for the revised version of this instrument require further investigation.

**Conclusion:**

The ISQ-8 shows promise as a reliable and valid measure of interoception in adolescents and adults on the autism spectrum, but additional work is needed to examine its psychometrics in this population. A free online score calculator has been created to facilitate the use of ISQ-8 latent trait scores for further studies of autistic adolescents and adults (available at https://asdmeasures.shinyapps.io/ISQ_score/).

**Supplementary Information:**

The online version contains supplementary material available at 10.1186/s13229-021-00440-y.

## Background

A core feature of autism as characterized by the Diagnostic and Statistical Manual of Mental Disorders, 5th Edition (DSM-5; [1]) is differences in response to sensory stimuli, including hyper-reactivity (exaggerated response), hyporeactivity (reduced or absent response), and unusual interest in sensory aspects of the environment (commonly referred to as “sensory seeking”; [1]). Alterations in multisensory integration and broader sensation and perception are also commonly observed in persons on the autism spectrum [2–5]. To date, much of the literature on sensory function in autism has focused on exteroceptive senses (e.g., vision, audition, or somatosensation; [6–8]). More recently, however, researchers have also begun to examine whether autism may be associated with differences in interoception, the processing of internal stimuli such as heartbeats and gut distention [9–11].

Interoception can be understood as the sense of the internal state of the body and contributes to allostasis by providing information about visceral processes (e.g., the perception of hunger, pain, temperature, thirst, or a number of other sensations; [12–14]. Interoceptive signals have also been suggested as a physiological substrate of emotional experience [15–17], and disrupted interoception has been implicated in the pathophysiology of multiple psychiatric conditions, including autism [9–11]. Poor interoceptive ability has specifically been hypothesized as the psychophysiologic basis of alexithymia [18–21], a personality trait that is commonly observed in the autistic^1^ population and characterized by difficulties with the identification or interpretation of one’s own or others’ emotional states [25, 26]. The study of interoception in autism, thus, has the potential to inform our understanding of not only sensory processing alterations, but also a number of affective features frequently reported in this population, such as alexithymia and emotion regulation difficulties [27].

Garfinkel et al. [28] put forth a comprehensive theoretical framework for conceptualizing interoception, proposing three separable dimensions of interoceptive experience: interoceptive accuracy, interoceptive sensibility, and interoceptive awareness. Interoceptive accuracy is defined as objective accuracy in detecting internal bodily sensations (e.g., can one accurately report when one’s heart is beating). Interoceptive sensibility is defined as self-perceived dispositional tendency to be internally self-focused and interoceptively cognizant (e.g., measured by self-report questions such as, “To what extent do you believe you focus on and detect internal bodily sensations?”; [16]). Interoceptive awareness is defined as metacognitive awareness of interoceptive accuracy (e.g., the accuracy of one’s subjective evaluation of one’s own ability to count heartbeats). It is important to note that interoceptive accuracy is most often tested through empirical measures of perception with an objective “ground truth” (e.g., heartbeat detection tasks; [29–32]), whereas interoceptive sensibility and awareness are subjective and, thus, typically tapped via self-report measures. It is also relevant to note that reports of interoceptive awareness do not always correlate strongly with ratings of interoceptive sensibility or performance on interoceptive accuracy tasks [28]. This finding does not necessarily that the construct of interoception is invalid; rather, it suggests that multiple facets of interoception exist, each contributing different yet meaningful information to our overall understanding of this construct.

When assessing whether autism is associated with differences in interoceptive accuracy or sensibility, investigators have often obtained seemingly discrepant results between empirical and self-report measures. Some studies have found increased interoceptive sensibility in people on the autism spectrum versus neurotypical peers [33], whereas others have found the opposite [21, 34, 35] or failed to detect between-group differences [25]. A similar pattern of discrepant results has been obtained for differences in interoceptive accuracy [33, 36, 37]. These findings provide additional evidence to suggest that the three facets of interoception are not interchangeable when determining whether a clinical population has impaired interoceptive ability. The discrepancies across studies may also be explained by limitations of the measures being used, highlighting the need for better tools that have been comprehensively, psychometrically evaluated.

One reason the results of extant studies may be so varied is because of how interoceptive sensibility has been conceptualized in self-report measures. Although different measures of interoceptive sensibility aim to assess the same latent construct, correlations between these measures are often modest [38, 39]. The low convergent validity between such measures suggests that the overlap in the constructs being assessed by different questionnaires may be quite minimal. For example, scores on the Multidimensional Assessment of Interoceptive Awareness (MAIA; [40]) have relatively weak correlations with other scales that purport to measure the same construct, including the Body Awareness Scale and the Interoception Sensory Questionnaire (*r*s < 0.35; [38, 39]). Moreover, though these rating scales are based on theoretical models, there is generally a lack of psychometric work validating these measures, particularly in the clinical populations about which they are so often used to make inferences. By providing theory-based definitions of interoceptive constructs, measures developed to date have allowed us to refine our conceptualization of interoception and to gather preliminary data from clinical populations. However, research in this field would benefit greatly from systematic psychometric analyses in large samples, particularly within clinical groups of interest. Thus, in the current study, we complement the aforementioned theory-driven approach by quantitatively assessing the statistical properties of a promising measure of interoceptive sensibility, the Interoception Sensory Questionnaire (ISQ).

The ISQ was developed by Fiene and colleagues [38] as a research tool intended specifically to assess the differences in interoceptive sensations between individuals with and without autism. The authors of this instrument qualitatively analyzed the content of online video blogs and semi-structured interviews with adults on the autism spectrum, drafting a preliminary 60-item questionnaire that was further reduced based on empirical analyses. In brief, the authors of the ISQ tested each item on its ability to discriminate between individuals with high and low levels of autistic traits, excluding 30 items that did not exhibit at least moderate between-group differences (*η*^2^ > 0.06). An exploratory factor analysis of the remaining 30 items (principal axis factoring of Pearson correlations) indicated that a single factor was sufficient to explain the covariance between item responses. A further 10 items were removed from the measure based on their low factor loadings (< 0.63), leaving 20 items in the final self-report tool. The final, 20-item version of the ISQ from showed high internal consistency and adequate convergent/discriminative validity. Due to the manner in which items were selected, the ISQ total score necessarily differentiated between autistic and neurotypical participants quite strongly. Notably, however, due to the relatively small autism sample in this study (*n* = 52), the authors were unable to confirm the factor structure of the ISQ specifically within the population of autistic adults.

A potential concern with the 20-item instantiation of the ISQ is redundancy in item content, as the questionnaire contains several pairs of items that seem to be “asking the same question twice” [41] (e.g., “Sometimes I don’t know how to interpret sensations I feel within my body” and “I find it difficult to read the signs and signals within my body [e.g., when I have hurt myself or need rest]”). Although some questionnaires include redundant item pairs in order to detect inconsistent responses, the authors of the ISQ made no mention of this in their original paper, indicating that item redundancy on this form was not intentional. Notably, when combined together into a total scale score, such redundant item pairs can cause a number of issues with an assessment. First, redundant items over-weight certain questions when deriving scores, as the content tapped by both items is effectively counted twice. Additionally, redundant items violate the assumption of local independence needed to conduct factor analysis. This can cause factor loadings and reliability coefficients to be artificially inflated and introduce bias [42–45].

Building on the work of Fiene et al. [38], this study aims to examine the psychometric properties of the ISQ in a larger sample of adults diagnosed with autism than previously tested, evaluating the fit of the proposed factor structure in the measure’s target population using confirmatory factor analysis (CFA). Furthermore, we seek to identify and eliminate any redundant items from the measure, producing a shortened form that satisfies the assumption of local independence. This reduced form will be tested in an item response theory (IRT) framework and tested for differential item functioning (DIF) across different sociodemographic groups. Lastly, we will investigate whether the ISQ is valid for use in self-reporting autistic adolescents, testing for the presence of DIF between adolescents and adults in our sample. We hypothesize that the unidimensional structure will remain intact, that several items can be removed, and that the items will function equivalently across sociodemographic groups, including between adolescents and adults.

## Methods

### Participants

This study was a secondary analysis of the ISQ completed by 495 adults and 187 adolescents on the autism spectrum recruited from the Simons Powering Autism Research Knowledge cohort (SPARK; [46]) using the SPARK Research Match service. These participants were recruited as a part of a larger study on the genetic underpinnings of sensory aspects of autism (RM0035Woynaroski). Participants were included if they submitted a genetic sample to SPARK, agreed to be contacted about further research, indicated reading proficiency in English, and were 13 years of age or older. Exclusion criteria included a diagnosed genetic disorder concomitant with autism (e.g., fragile X syndrome), or significant sensory impairments (i.e., blindness and/or deafness). The full sample was 51.6% male, 82.2% non-Hispanic White, and had a mean age of 31.2 years (range: 13.1–77.8 years). Full demographic information for the sample and adolescent/adult subsamples can be found in Table 1. All participants gave informed consent or assent for participation in the study, and parental consent was obtained for minors. All study procedures were approved by the Institutional Review Board at Vanderbilt University Medical Center.

**Table 1:** Demographics for adult, adolescent, and combined samples

	Adults	Adolescents	Combined
*n*	495	187	682
Age in years (*M* [*SD*])	37.2 (13.3)	15.4 (1.4)	31.2 (14.9)
Sex (M/F)	206/289	146/41	352/330
*Gender*			
Cisgender male	194	138	332
Cisgender female	255	39	294
Transgender male	5	0	5
Transgender female	1	0	1
Other/non-binary	20	2	22
*Race*^a^			
White	433 (87.5%)	168 (89.8%)	601 (88.1%)
Asian	19 (3.8%)	3 (1.6%)	22 (3.2%)
Black/African American	22 (4.4%)	12 (6.4%)	34 (5.0%)
Native American	28 (5.7%)	5 (2.7%)	33 (4.8%)
Native Hawaiian	3 (0.6%)	3 (1.6%)	6 (0.9%)
Other	20 (4.0%)	3 (1.6%)	23 (3.4%)
Hispanic/Latino Ethnicity	37 (7.5%)	15 (8.0%)	52 (7.6%)
*Annual household income (USD)*			
≤ $20,000	138 (27.9%)	18 (9.6%)	156 (22.9%)
$21,000–$35,000	74 (14.9%)	29 (15.5%)	103 (15.1%)
$36,000–$50,000	60 (12.1%)	29 (15.5%)	89 (13.0%)
$51,000–$65,000	40 (8.1%)	17 (9.1%)	57 (8.4%)
$66,000–$80,000	43 (8.7%)	27 (14.4%)	70 (10.3%)
$81,000–$100,000	34 (6.9%)	19 (10.2%)	53 (7.8%)
$101,000–$130,000	32 (6.5%)	23 (12.3%)	55 (8.1%)
$131,000–$160,000	19 (3.8%)	11 (5.9%)	30 (4.4%)
≥ $161,000	23 (4.6%)	6 (3.2%)	29 (4.3%)
ISQ total score (*M* [*SD*])	61.9 (30.5)	53.1 (27.3)	59.5 (29.9)
ISQ-8 Latent trait score (*M* [*SD*])	0.06 (0.95)	− 0.16 (0.90)	0.00 (0.94)

ISQ, Interoception Sensory Questionnaire; ISQ-8, 8-item ISQ short form derived in this study

^a^Percentages for race exceed 100%, as individuals could select more than one option

### Procedures

Participants for the study were recruited as a part of the SPARK Research Match Process. Briefly, individuals enrolled in SPARK and meeting inclusion/exclusion criteria for the larger study on the genetic basis of sensory alterations in autism (RM0035Woynaroski) were contacted about participation in a supplemental research opportunity via email. Interested individuals subsequently consented for participation and completed a series of surveys regarding their sensory experiences, including the ISQ, via an online platform. Demographics were drawn from the larger SPARK study.

### Measures

The ISQ [38] is a 20-item self-report questionnaire intended to measure interoceptive challenges in autistic adults using a single factor scale. The items aim to identify the broad ways in which individuals on the autism spectrum may experience differences in interoceptive processing using a 7-point Likert scale (1 = “Not true at all of me”, 7 = “Very true of me”) where a higher score indicates more difficulty registering or interpreting interoceptive sensations. Three items were reverse-scored to maintain scoring consistency.

The reliability of the ISQ in autistic individuals, as estimated by Cronbach’s alpha, is quite high, both in the sample reported by Fiene et al. (*α* = 0.96) and the current sample of adults on the autism spectrum (*α*  = 0.96, 95% CI [0.95, 0.97]). Fiene et al. [38] found evidence for the questionnaire’s construct validity as evidenced by associations between the ISQ, the Toronto Alexithymia Scale [47], Big Five personality traits [48], and subscales from the MAIA [40]. Specifically, alexithymia scores from the Alexithymia Scale had a strong positive correlation with interoceptive difficulty as measured by the ISQ. Extraversion, body listening, emotional awareness, attention regulation, and self-regulation were all inversely correlated with interoceptive difficulty. Further correlational analyses showed that gender, age, and years of education were not associated with ISQ scores in a neurotypical group of 459 participants [38].

### Statistical analysis

#### Descriptive statistics

All statistical analyses were conducted in the R programming environment [49]. Item-level descriptive statistics including item means and standard deviations, and skewness were calculated. In addition, we analyzed the polychoric item correlation matrix, examining the magnitude of correlations between each item and all other items on the ISQ as a measure of item redundancy [50]. The mean (polychoric) correlation between each item and all other items, as well as the number of intercorrelations for each item exceeding 0.7, was reported. As correlations of 0.7 reflect approximately 50% shared variance between the latent continua underlying each item pair, correlations above this value are highly suggestive of item content redundancy [50].

#### Confirmatory factor analysis

Confirmatory factor analysis (CFA) was used to fit the one-factor model proposed by Fiene et al. [38] in our sample of autistic adults in order to determine whether the ISQ conforms to a unidimensional structure in this population. We fit the model using a Diagonally Weighted Least Squares estimator [51] with a mean- and variance-corrected test statistic (i.e., “WLSMV” estimation), as implemented in the R package *lavaan* [52]. As very few of the item responses in our dataset contained missing values (0.004% missing item responses), we handled missing values in our model using pairwise deletion.

Model fit was evaluated using the chi-square test of exact fit. However, given the test’s high likelihood of rejecting models that differ trivially from the population structure (cf. [53]), several additional fit indices were also calculated, including the comparative fit index (CFI; [53]), Tucker-Lewis index (TLI; [54]), root mean square error of approximation (RMSEA; [55]), standardized root mean square residual (SRMR; [56]), correlation root mean square residual (CRMR; [57]), and weighted root mean square residual (WRMR; [58, 59]). Notably, we employed the categorical maximum likelihood (cML) estimators of the CFI, TLI, and RMSEA proposed by Savalei [60], as these indices better approximate the population values of the maximum likelihood-based fit indices used in linear CFA. Moreover, the SRMR and CRMR were calculated using the unbiased estimators (i.e., SRMR_u_ and CRMR_u_) proposed by Maydeu-Olivares [57, 61] and implemented in *lavaan* for categorical estimators. We judged fit using the widely accepted guidelines of Hu & Bentler [56], which state that CFI/TLI values of > 0.95, SRMR (and by extension CRMR) values of < 0.08, and RMSEA values of < 0.06 indicate good model fit (though see [62–64] for limitations of standardized fit index cutoffs). Though the WRMR is a less well-studied index of fit, recent simulation work supports the assertions of Yu [59] that values below 1.0 generally suggest good model fit [58].

In addition to global fit indices, we checked for localized areas of model misfit using the approach proposed by Saris et al. [65]. In this approach, the modification index (MI) of a structural coefficient is considered alongside the expected parameter change and the power of the MI test to determine whether two items likely exhibited correlated error terms (as determined by an expected parameter change of ≥ 0.1). Information from this analysis and the analysis of inter-item correlations was combined to determine whether any items on the scale should be deemed redundant and eliminated. A model-based estimate of internal consistency reliability, McDonald’s [66] coefficient omega (*ω*), was calculated from the one-factor model using the categorical data estimator proposed by Green and Yang [67]. 95% confidence intervals for omega were constructed using the bias-corrected and accelerated bootstrap approach (1000 resamples) recommended by Kelley and Pornprasermanit [68].

### Item reduction

Using the information from the misspecification analysis and correlation matrix inspection, the set of items was reduced to the maximum number of items that satisfied the following criteria: (a) no polychoric correlation between two items exceeds 0.7 and (b) the Saris et al. [65] method does not flag any item pair as having correlated error terms with an estimated parameter change (EPC) of 0.1 or greater. The reduced scale was re-fit using the same CFA methods, and its fit was compared to that of the longer form.

### Item response theory analysis

After reducing the number of items on the ISQ, we analyzed the resulting short form within an item response theory framework, fitting data from those items to a unidimensional graded response model [69] in our adult sample. The model was fit using maximum marginal likelihood estimation via the Bock–Aitkin EM algorithm [70], as implemented in the *mirt* R package [71]. Model fit was assessed using the limited-information *C*_2_ statistic [72, 73], as well as *C*_2_-based approximate fit indices and SRMR. The guidelines for adequate fit proposed by Maydeu-Olivares and Joe [74] for the RMSEA_2_ and SRMR were used to establish adequate fit of the IRT model. To further confirm that item redundancy was not affecting IRT parameters, we calculated Chen and Thissen’s [75] standardized local dependency (LD) *χ*^2^ statistic for each item pair. Standardized LD-*χ*^2^ values greater than 10 are typically indicative of practically significant local dependence [76].

Once the adequacy of the model was established, we used information generated by the IRT parameters to further understand the psychometrics of the shortened ISQ form. Marginal reliability of the latent trait score was calculated, and the 95% confidence interval for this value was constructed using a simple percentile bootstrap (1000 resamples). Reliability coefficients for each individual respondent were also examined, with values greater than 0.7 being deemed sufficiently reliable for interpretation at the individual level. The performance of each item was also evaluated by examining item characteristic curves and item information curves, as well as testing for differential item functioning (DIF). Items were evaluated for DIF in the adult sample across groups based on age (> 40 vs. ≤ 40 years), biological sex, gender identity, and annual household income (> $50,000 vs. ≤ $50,000). Age and income cut-points were chosen based on approximate median splits. DIF by race/ethnicity was not able to be tested due to the small number of individuals identifying as categories other than non-Hispanic White. DIF was tested using the iterative Wald test procedure proposed by Cao et al. [77] and implemented by Williams [78], with *p* values < 0.05 (FDR-corrected; [79]) used to flag items for DIF. Significant omnibus Wald tests were followed up with tests of individual item parameters to determine which parameters significantly differed between groups.

In order to test the validity of the shortened ISQ in a population of adolescents on the autism spectrum, we fit a multiple-group graded response model to data in both the adolescent and adult samples, assessing overall model fit using the criteria described above. To determine whether scores in the two groups were comparable, we tested for DIF between adolescents and adults using the iterative Wald test procedure [77, 78] and an FDR-corrected *p-*value threshold of 0.05. As no significant DIF was found between the groups, we then re-fit the graded response model to the full dataset, using item parameters from this final model to calculate latent trait scores on the ISQ. Lastly, to examine the effects of demographics on ISQ latent trait scores, we then regressed the ISQ latent trait score on age (in years), sex (male vs. female), and the interaction between age and sex.

## Results

### Descriptive statistics

ISQ means, standard deviations, skewness, number of large correlations (*r* > 0.7), and mean correlations are displayed in Table 2. Several items (Items 6, 10, 11, 12, 13, 14, 16, 18) showed many (> 5) large correlations (> 0.7). Out of 190 unique correlations, there were 43 (22.6%) that were greater than 0.7, indicating that there was likely a high degree of item content overlap [50]. Several problematic item pairs (e.g., Item 5. *I find it difficult to describe feelings like hunger, thirst, hot or cold* and Item 13. *It is difficult for me to describe what it feels like to be hungry, thirsty, hot, cold or in pain*; Item 3 *I have difficulty feeling my bodily need for food* and Item 11. *I have difficulty understanding when I am hungry or thirsty*; Item 10. *I find it difficult to read the signs and signals within my own body (e.g., when I have hurt myself or I need to rest)* and Item 14. *I am confused about my bodily sensations*) had a very high degree of correlation (e.g., *r*_poly_ = 0.85 for Items 5 and 13).

**Table 2:** ISQ item content and descriptive statistics for adult sample

Item content	*N*	*M*	*SD*	Skew	*N*_*r*>0.7_	$\overline{r }$
**1. I have difficulty making sense of my body’s signals unless they are very strong**	444	3.86	1.99	0.01	3	0.60
2. I tend to rely on visual reminders (e.g. times on the clock) to help me know when to eat and drink	444	3.61	2.18	0.20	0	0.47
**3. I have difficulty feeling my bodily need for food**	444	3.38	2.19	0.34	4	0.64
**4. I’m not sure how my body feels when it’s a hot day**	444	2.25	1.81	1.30	0	0.52
**5. I find it difficult to describe feelings like hunger, thirst, hot or cold**	443	2.6	1.97	1.00	4	0.64
6. Sometimes I don’t know how to interpret sensations I feel within my body	444	3.88	2.02	0.02	7	0.65
7. If I injure myself badly, even though I can feel it, I don’t feel the need to do much about it	443	3.04	2.09	0.59	0	0.50
8. I only notice I need to eat when I’m in pain or feeling nauseous or weak	444	3.17	2.21	0.53	3	0.61
**9. There are times when I am only aware of changes in my body because of the reactions of other people**	444	3.05	1.97	0.55	1	0.61
10. I find it difficult to read the signs and signals within my own body (e.g. when I have hurt myself or I need to rest)	444	3.23	2.01	0.43	12	0.70
11. I have difficulty understanding when I am hungry or thirsty	443	2.88	2.01	0.69	7	0.65
**12. I find it difficult to identify some of the signals that my body is telling me (e.g. If I’m about to faint or I’ve over exerted myself)**	443	3.27	2.03	0.43	6	0.65
13. It is difficult for me to describe what it feels like to be hungry, thirsty, hot, cold or in pain	442	2.74	1.97	0.85	8	0.67
14. I am confused about my bodily sensations	443	3.06	1.93	0.55	8	0.66
**15. I have difficulty locating injury in my body**	444	2.45	1.8	1.09	2	0.60
16. Sometimes, when my body signals a problem, I have difficulty working out what the problem might be	443	3.35	1.93	0.36	8	0.66
17. I don’t tend to notice feelings in my body until they’re very intense	443	3.31	2.09	0.44	4	0.66
18. I find it difficult to put my internal bodily sensations into words	443	3.58	2.15	0.26	5	0.64
19. Even when I know that I am hungry, thirsty, in pain, hot or cold, I don’t feel the need to do anything about it	444	2.91	1.89	0.62	1	0.59
**20. Even when I know that I am physically uncomfortable, I do not act to change my situation**	444	3.01	1.88	0.53	1	0.55

Total *n* in the adult sample is 495. *N*_*r*>0.7_, the number of items with which a given item correlates with a strength greater than 0.70; $\stackrel{-}{{\varvec{r}}}$, average inter-item correlation. ISQ-8 items are bolded

### Confirmatory factor analysis

Model fit for the 20-item ISQ was inadequate based on conventional fit criteria (Table 3). The Chi-square test was significant (*p* < 0.001), rejecting the null hypothesis of exact model fit. Other fit indices also failed to meet a priori cutoff values (i.e., CFI_cML_/TLI_cML_ > 0.95, RMSEA_cML_ < 0.06, WRMR < 1.0, and SRMR_u_/CRMR_u_ < 0.08), suggesting that this model did not fit the data in our sample well. Using McDonald’s omega, the model showed good reliability (*ω* = 0.966, 95% bootstrapped CI [0.961, 0.971]); however, as a model-based reliability coefficient is only as valid as the model it is based on [80], this coefficient should be interpreted with caution given the poor fit of the model. Factor loadings for the items in the CFA model are displayed in Table 4.

**Table 3:** Fit indices for original and revised ISQ confirmatory factor models

ISQ Version	χ2	*df*	*p* value	CFI_cML_	TLI_cML_	RMSEA_cML_ [90% CI]	SRMR_u_ [90% CI]	CRMR_u_ [90% CI]	WRMR	*ω*
ISQ-20	1536.18	170	< 0.001	0.838	0.819	0.141 [0.135, 0.148]	**0.057** [0.049, 0.061]	**0.058** [0.052, 0.064]	1.765	0.966
ISQ-8	46.30	20	0.007	**0.984**	**0.977**	0.063 [0.042, 0.085]	**0.019** **[0.012, 0.025]**	**0.021** **[0.014, 0.028]**	**0.499**	0.901

*χ*^2^ based on limited-information WLSMV estimator. Values in bold exceed standard fit index cutoffs

CFI_cML_, comparative fit index (based on categorical maximum likelihood estimator); TLI_cML_, Tucker-Lewis index (based on categorical maximum likelihood estimator); RMSEA_cML_, root mean square error of approximation (based on categorical maximum likelihood estimator); SRMR_u_, standardized root mean square residual (unbiased estimator); CRMR_u_, correlation root mean square residual (unbiased estimator); WRMR, weighted root mean square residual; *ω*, coefficient omega (internal consistency reliability)

**Table 4:** Factor loadings for ISQ-20 and ISQ-8

Item	ISQ-20		ISQ-8	
	Factor loading	Communality	Factor loading	Communality
1	0.763	0.583	0.757	0.573
2	0.606	0.367		
3	0.833	0.693	0.781	0.609
4	0.673	0.453	0.715	0.511
5	0.852	0.726	0.829	0.687
6	0.867	0.751		
7	0.651	0.424		
8	0.789	0.622		
9	0.792	0.628	0.801	0.642
10	0.890	0.792		
11	0.866	0.750		
12	0.837	0.701	0.842	0.710
13	0.886	0.785		
14	0.887	0.787		
15	0.777	0.604	0.773	0.598
16	0.877	0.769		
17	0.839	0.705		
18	0.837	0.701		
19	0.776	0.602		
20	0.717	0.514	0.673	0.453

### Item reduction and short form construction

Misspecification analysis was conducted to identify the specific pairs of items driving the misfit of the unidimensional model. Based on this method, several pairs of items were found to have omitted error correlations (i.e., EPC > 0.1; [51]), indicating item content redundancy (e.g., Items 19/20, 5/13, and 3/11; see Additional file 1: Table S1 for a full list of flagged item pairs) sing the polychoric correlation matrix, the items were ordered by number of large correlations (> 0.7). First, the 6 items with the most intercorrelations were removed (Items 6, 10, 11, 13, 14, 16). Item 17 was then cut because of its high correlations with Items 12 and 1 (*r* values = 0.73 and 0.71, respectively; 17. *I don’t tend to notice feelings in my body until they’re very intense*; 12. *I find it difficult to identify some of the signals that my body is telling me [e.g., If I’m about to faint or I’ve over exerted myself]*; 1. *I have difficulty making sense of my body’s signals unless they are very strong*). After these reductions, several large correlations were still present among the 13 remaining items. To further reduce item redundancy, each of the flagged item pairs was compared, and the item whose content was more general was retained for the final scale. Using this criterion, Item 3 was kept over Item 8 (3. *I have difficulty feeling my bodily need for food*; 8. *I only notice I need to eat when I’m in pain or feeling nauseous or weak*), Item 20 was kept over Item 19 (20. *Even when I know that I am physically uncomfortable, I do not act to change my situation*; 19. *Even when I know that I am hungry, thirsty, in pain, hot or cold, I don’t feel the need to do anything about it*), and item 5 was kept over Item 18 (5. *I find it difficult to describe feelings like hunger, thirst, hot or cold*; 18. *I find it difficult to put my internal bodily sensations into words*). This item reduction process resulted in a 10-item scale with all inter-item correlations less than 0.7. Based on information from the misspecification analyses item pairs 2/3 (2. *I tend to rely on visual reminders (e.g., times on the clock) to help me know when to eat and drink*; 3. *I have difficulty feeling my bodily need for food*) and 7/20 (7. *If I injure myself badly, even though I can feel it, I don’t feel the need to do much about it*; 20. *Even when I know that I am physically uncomfortable, I do not act to change my situation*) were further identified as misspecified, and Items 3 and 20 were retained due to their more general content. The final short form of the ISQ contained 8 items (ISQ Items 1, 3, 4, 5, 9, 12, 15, and 20; Additional file 1: Table S2).

The short form ISQ (ISQ-8) showed far better fit after item reduction using the same criteria (Table 3). The Chi-square test once again rejected the null hypothesis of exact model fit (*p* = 0.007), signaling at least some degree of model misspecification. Other fit indices met a priori criteria (i.e., CFI_cML_/TLI_cML_ > 0.95, RMSEA_cML_ < 0.06, WRMR < 1.0, and SRMR_u_/CRMR_u_ < 0.08), demonstrating trivial levels of global misfit, and misspecification analysis of this reduced-item set showed no flagged pairs, indicating a low likelihood of item content redundancy. Reliability of the model was evaluated with coefficient omega (*ω* = 0.901, 95% bootstrapped CI [0.886, 0.913]) suggesting good internal consistency for this 8-item model.

### Item response theory analyses

The model for the ISQ-8 showed overall good fit in the adult sample (*C*_2_(20) = 32.5, *p* = 0.038, *CFI*_C2_ = 0.997, *RMSEA*_C2_ = 0.036, *SRMR* = 0.040). Additionally, the standardized LD-χ^2^ values were all less than 5.79, providing no evidence for remaining item redundancies. The marginal reliability of the ISQ-8 was good (*ρ*_xx_ = 0.891, 95% bootstrapped CI [0.881, 0.890]), further demonstrating the psychometric adequacy of the reduced scale. Scores for individual participants all had reliability values greater than 0.7, indicating the 8-item form measured the construct with sufficient precision in all cases. Factor loadings and IRT slope/intercept parameters can be found in Table 4.

Based on an examination of the item category characteristic curves (Additional file 1: Figure S4), we concluded that a 7-point response scale was not optimal for the ISQ-8. For all 8 items, the plots showed that there were item responses that at no point on the latent continuum were the most probable choice, thus suggesting that there were too many response options. As a result, item responses were collapsed together to create a 5-point scale (i.e., the “2”/“3” responses were combined together into a single response option, as were the “5”/ “6” responses). Using this new 5-point scale, the IRT model was re-run in the adult sample. This model also showed good fit (*C*_2_(20) = 32.0, *p* = 0.043, CFI_C2_ = 0.997, RMSEA_C2_ = 0.035, SRMR = 0.038), no local dependencies (LD-*χ*^2^ values < 9.26), and good reliability (*ρ*_xx_ = 0.887, 95% bootstrapped CI [0.878, 0.897]). EAP-estimated latent trait scores derived from the recoded ISQ-8 correlated very highly with those derived from the original ISQ-8 (*r* > 0.997). The item trace lines for the 5-point scale indicated more consistent response utilization than those for the 7-point scale, but the middle response was still shown to be underutilized in a number of cases (Additional file 1: Figure S4).

Differential item function was also evaluated using the iterative Wald test procedure to identify differences in performance by age, sex, gender, and household income. No differential item functioning was found between any of the tested groups on any item (all *p*’s > 0.101, FDR corrected; see Additional file 1: Table S3 for full DIF results). Given that no difference was observed between the adult and adolescent groups, the two were combined and run together in another model using the 5-point scale. This model showed good overall fit (*C*_2_(20) = 48.2, *p* < 0.001, *CFI*_C2_ = 0.994, *RMSEA*_C2_ = 0.046, *SRMR* = 0.036), no local dependence (LD-*χ*^2^ values all < 9.14), and good reliability (*ρ*_xx_ = 0.880, 95% bootstrapped CI: [0.871, 0.889]). Latent trait scores from this model (EAP estimation) correlated very highly with total scores on the original ISQ-20 (*r* = 0.942). We, therefore, concluded that this short form adequately represented the longer measure from which it was derived. A regression of ISQ-8 score on age and sex across the full sample explained very little of the variance in interoceptive sensibility (*R*^2^ = 0.045), although a statistically significant main effect of sex indicated moderately higher levels of interoceptive difficulties in autistic women and girls compared to autistic men and boys (*β*_F-M_ = 0.612, *p* < 0.001). The main effect of age and the age by sex interaction were not significant (*p*’s > 0.104). These results were found to be the same according to both reported sex or gender identity.

## Discussion

The current study is the first to evaluate the latent structure of an interoceptive sensibility questionnaire in a large sample of autistic individuals, presenting preliminary data to support the use of a shortened version of the ISQ (ISQ-8) in this population. The unidimensional factor model of the full-length ISQ proposed by Fiene and colleagues [38] exhibited suboptimal fit to the data in our sample, likely driven by a large number of unmodeled correlated error terms. However, after removing a number of redundant items and reducing the number of response options from 7 to 5, we were able to create a psychometrically-improved version of the ISQ with unidimensional structure, excellent model-data fit, trivial levels of misspecification, and high score reliability. The ISQ-8 items did not function differently across sociodemographic groups, and the lack of DIF seen between adolescent and adult samples supports the validity of this measure in adolescents on the autism spectrum in addition to autistic adults. Although scores on the ISQ-8 were independent of age, we did find moderately higher levels of interoceptive difficulties in autistic females. This finding notably differed from the lack of ISQ score differences by gender found in the original study by Fiene et al. [38], potentially indicating a sex difference that is unique to individuals on the autism spectrum. Although further validation of the ISQ-8 is needed in both autism and neurotypical samples, our study provides a necessary first step toward developing a robust self-report measure of interoceptive sensibility in the autistic population.

Though Feine et al. [27] reported that the original ISQ form was unidimensional in structure, the fit of our one-factor CFA model was inadequate, driven by the psychometric consequences of doublet factors (i.e., “asking the same question twice”; [41, 81]. Item pairs, such as ISQ items 5 (*I find it difficult to describe feelings like hunger, thirst, hot or cold*) and 13 (*It is difficult for me to describe what it feels like to be hungry, thirsty, hot, cold or in pain*) correlated extremely highly, reflecting shared variance due to the latent factor and additional shared variance due to overlap in item wording or semantic content. When not accounted for in a given model, item redundancy can artificially inflate factor loadings, IRT slope parameters, and model-based reliability coefficients [42–45], causing some authors to favor high item inter-correlations over the broader content coverage needed for an instrument to have construct validity [50]. Furthermore, as the use of a measure’s summed total score implies a latent trait model with uncorrelated errors [82], questionnaires such as the ISQ-20 with many redundant items produce total scores that are biased estimates of the underlying latent trait. Thus, in order to improve the psychometric adequacy of the ISQ, we felt justified in removing many of the questionnaire’s items to meet the assumption of local dependence.

Item response theory models were then fit to the reduced form, confirming its unidimensionality, good reliability, and lack of local dependence. However, analysis of item trace lines demonstrated that the 7-point response scale originally proposed by Fiene contained more response options than meaningfully used by autistic participants. We thus re-coded the item responses along a 5-point scale, reducing the amount of between-subject error variance attributable to trait-unrelated tendencies to respond closer to the middle of a bipolar scale. Although item trace lines after re-coding indicated that the middle item response was still underutilized in most cases (see also [83] for an argument against the use of neutral response options), it is possible that this pattern would not be observed if participants were to respond to ISQ-8 items on a 5-point scale rather than a recoded 7-point scale. Thus, while this finding does provide preliminary support for the possible elimination of a neutral response option in future versions of the ISQ (see also: [72]), further research using the 5-point response scale is necessary to make conclusive recommendations.

After confirming the psychometric adequacy of the ISQ-8 in our sample of autistic adults, we tested the factorial validity of the ISQ-8 in our adolescent sample. Our DIF analyses found that all ISQ-8 items functioned equivalently between adults and adolescents on the autism spectrum, supporting the decision to derive item parameters from a combined adolescent-adult sample. Although model fit was slightly reduced when compared to the adult-only sample (i.e., the *C*_2_-based RMSEA increased slightly), the unidimensional graded response model fit this data adequately, justifying the interpretation of estimated ISQ-8 latent trait scores in both adolescents and adults on the autism spectrum. To facilitate the use of these latent trait scores in future studies, we have created an easy-to-use online scoring tool that can convert patterns of ISQ-8 item responses (on either a 5- or 7-point scale) into calibrated latent trait estimates and corresponding T-scores (available at https://asdmeasures.shinyapps.io/ISQ_Score/). However, as these scores have only been validated in autistic adolescents and adults, future studies are necessary to validate these scores in adolescents and adults *without* autism diagnoses and to determine whether DIF exists between participants on the autism spectrum and the general population.

This work has meaningful implications for the study of interoception in autistic people, as it provides strong psychometric support for the use of the ISQ-8 as a measure of interoceptive sensibility in this population. While research to date has demonstrated broad group differences in interoceptive constructs associated with autism, the lack of validation in many forms of measurement makes it challenging to identify exactly where these differences lie. The value of psychometric work on the ISQ specifically is that researchers can now employ this tool to examine how interoceptive traits manifest in persons on the autism spectrum, knowing that differences in interoceptive sensibility across this population are not driven by qualitatively different item responding across sociodemographic groups. This measure can also be used to test the convergent validity of other interoceptive sensibility questionnaires in the autistic population, allowing future research to identify whether other tools such as the Body Perception Questionnaire (BPQ; [81]) and MAIA are tapping similar interoceptive constructs in the autistic population. Perhaps most importantly, this work builds on the foundational work of Fiene et al. [27] to provide a robust measurement tool for use in autism interoception research, setting the stage for future investigations of the relations between self-reported interoceptive differences, autistic features, and co-occurring psychopathology.

This study had several notable strengths including its sample size, robust statistical analyses, inclusion of adolescents in the sample, and ability to test the psychometric properties of a measure within a specific clinical group of interest. Psychometric studies are crucial to the success of research in psychology, as the inferences that we can make about psychological constructs are limited by the validity of the tools used to measure them [84]. Given the large sample available through SPARK, we were able to test the psychometric properties of the ISQ in its target population, using that information to refine and validate the scale in both adolescents and adults on the autism spectrum. In our sample, the final form of the ISQ-8 demonstrates high reliability, unidimensionality, and a lack of item redundancy. This brief questionnaire has excellent psychometric properties in autistic individuals, and future studies will determine whether the ISQ-8 is suitable to quantify interoceptive sensibility in other psychiatric conditions thought to be associated with interoceptive deficits [10].

## Limitations

One major limitation of this study is the lack of neurotypical individuals with whom to compare broad group differences or conduct differential item functioning analyses by diagnosis. Without this comparison, it is difficult to conclude how individuals with and without autism differ on the ISQ, and it remains possible that the diagnostic group differences observed by Fiene et al. were significantly distorted by DIF. It is also worth noting that our sample contained a relatively high proportion of female participants compared to estimates in the wider autism population (currently estimated at a 3:1 male to female ratio in research; [85]). Our finding that interoception may differ according to sex and gender is in accordance with other work in autism research suggesting sex-based differences in exteroceptive sensory functioning (e.g., [86, 87]). Furthermore, while this study proposes a 5-response scale for the ISQ-8, our data were not collected using this method; thus, the psychometric properties for the 5-response instantiation of this instrument are not entirely known. Additionally, the ISQ-8 with a 5-point scale is not validated in neurotypical or other clinical groups where this form may be of interest. Therefore, though the present results support the recommendation that future versions of the ISQ use a 5-point response scale, further work is needed to assess the adequacy of this response format in both autistic and neurotypical populations.

Another shortcoming of this study is the lack of tests of convergent and broader nomological validity. The present study did not test whether the ISQ converged with other measures of interoceptive sensibility (e.g., the BPQ) or showed theoretically-supported associations with related constructs, such as core autism symptoms, anxiety, or neuroticism. This type of research is necessary in the future to determine whether the ISQ taps the same construct that other interoceptive sensibility measures aim to assess and whether this measure can predict important clinical outcomes such as affective symptoms or anxiety.

Lastly, it remains unknown whether self-rated interoceptive sensibility on the ISQ correlates meaningfully with measures of interoceptive accuracy or interoceptive awareness. This limitation in particular makes it challenging to understand how the ISQ is situated within the nomological network of the superordinate interoception construct. While there is some ambiguity regarding the degree to which separable interoceptive subconstructs should correlate, general difficulties in interoceptive ability should theoretically cause all three aspects of interoception to covary to some degree.

Another limitation of the SPARK pool is that autism diagnoses are self-reported and are not verified. Although web-based autism registries have been shown to be reliable [88], the lack of confirmation of autism diagnoses limits the study’s ability to draw definitive psychometric conclusions about the performance of the ISQ in this population. This study, therefore, begs for replication in a large sample of individuals for whom autism diagnoses are independently confirmed via gold-standard measures.

In sum, the limitations of this study include a lack of neurotypical control group, unrepresentative sample of the wider autistic population, reliance of our findings on data derived from the longer ISQ-20, and the lack of tests of the nomological validity of the ISQ-8. Future work would benefit from comparing autistic and neurotypical individuals with other neuropsychiatric conditions using the ISQ-8, particularly testing whether significant differential item functioning exists across groups. Furthermore, it would be valuable to compare the scores on this measure with other measures of interoceptive sensibility, interoceptive awareness, and interoceptive accuracy. Doing so would not only help establish a fuller picture of interoceptive differences in autism, but also advance our understanding of the psychometrics of the various tools intended to tap various aspects of interoception across populations.

## Conclusions

The ISQ is a recently developed measure intended to index interoceptive sensibility in autistic people. However, it has previously lacked robust psychometric evidence supporting its use when evaluating persons on the autism spectrum. Drawing upon data from a large sample obtained via partnership with SPARK, we sought to investigate the ISQ using CFA and proposed a new, short-form version (the ISQ-8) with superior psychometric properties for use in adolescents and adults on the autism spectrum. This revised questionnaire shows great promise as a tool for measuring interoceptive sensibility in autism going forward and would benefit from further studies testing its construct validity both within the autism population and across diagnostic groups.

## Supplementary Information

**Additional file 1**. Supplemental figures and tables.

## Data Availability

Approved researchers can obtain the SPARK population data set described in this study by applying at https://base.sfari.org. The remainder of research materials can be obtained from the corresponding author upon request.

## Abbreviations

ISQ: Interoception Sensory Questionnaire
DIF: Differential item functioning
BPQ: Body Perception Questionnaire
MAIA: Multidimensional assessment of interoceptive awareness
CFA: Confirmatory factor analysis
IRT: Item response theory
SPARK: Simons powering autism research knowledge cohort
MI: Modification Index
EPC: Estimated parameter change
LD: Local dependence

## Footnotes

1 The terms ‘autistic person’ and ‘person on the autism spectrum’ are the preferred language of the majority of people diagnosed as autistic [22–24]. Out of respect for these preferences, we use these terms to refer to individuals on the spectrum rather than exclusively using person-first language in the present manuscript.

## References

[1] American Psychiatric Association (2013). Diagnostic and statistical manual of mental disorders (DSM-5®).

[2] Cascio CJ, Woynaroski T, Baranek GT, Wallace MT (2016). Toward an interdisciplinary approach to understanding sensory function in autism spectrum disorder. Autism Res.

[3] Baum SH, Stevenson RA, Wallace MT (2015). Behavioral, perceptual, and neural alterations in sensory and multisensory function in autism spectrum disorder. Prog Neurobiol.

[4] Beker S, Foxe JJ, Molholm S (2018). Ripe for solution: delayed development of multisensory processing in autism and its remediation. Neurosci Biobehav Rev.

[5] Ben-Sasson A, Gal E, Fluss R, Katz-Zetler N, Cermak SA (2019). Update of a meta-analysis of sensory symptoms in ASD: a new decade of research. J Autism Dev Disord.

[6] Crane L, Goddard L, Pring L. Sensory processing in adults with autism spectrum disorders: Autism. 2009; Available from: https://journals.sagepub.com/doi/10.1177/1362361309103794. 10.1177/1362361309103794 19369385

[7] Hazen EP, Stornelli JL, O’Rourke JA, Koesterer K, McDougle CJ (2014). Sensory symptoms in autism spectrum disorders. Harv Rev Psychiatry.

[8] Robertson CE, Baron-Cohen S (2017). Sensory perception in autism. Nat Rev Neurosci.

[9] DuBois D, Ameis SH, Lai M-C, Casanova MF, Desarkar P (2016). Interoception in autism spectrum disorder: a review. Int J Dev Neurosci.

[10] Khalsa SS, Adolphs R, Cameron OG, Critchley HD, Davenport PW, Feinstein JS (2018). Interoception and mental health: a roadmap. Biol Psychiatry Cognit Neurosci Neuroimaging.

[11] Quattrocki E, Friston K (2014). Autism, oxytocin and interoception. Neurosci Biobehav Rev.

[12] Craig AD (2002). How do you feel? Interoception: the sense of the physiological condition of the body. Nat Rev Neurosci.

[13] Tsakiris M, Preester HD (2018). The interoceptive mind: from homeostasis to awareness.

[14] Kleckner IR, Zhang J, Touroutoglou A, Chanes L, Xia C, Simmons WK, et al. Evidence for a large-scale brain system supporting allostasis and interoception in humans. Nat Hum Behav. 2017;1. Available from: https://www.ncbi.nlm.nih.gov/pmc/articles/PMC5624222/. 10.1038/s41562-017-0069 PMC5624222 28983518

[15] Adolfi F, Couto B, Richter F, Decety J, Lopez J, Sigman M (2017). Convergence of interoception, emotion, and social cognition: a twofold fMRI meta-analysis and lesion approach. Cortex.

[16] Garfinkel SN, Critchley HD (2013). Interoception, emotion and brain: new insights link internal physiology to social behaviour. Soc Cogn Affect Neurosci.

[17] Seth AK (2013). Interoceptive inference, emotion, and the embodied self. Trends Cogn Sci.

[18] Trevisan DA, Altschuler MR, Bagdasarov A, Carlos C, Duan S, Hamo E (2019). A meta-analysis on the relationship between interoceptive awareness and alexithymia: distinguishing interoceptive accuracy and sensibility. J Abnorm Psychol.

[19] Murphy J, Catmur C, Bird G (2017). Alexithymia is associated with a multidomain, multidimensional failure of interoception: evidence from novel tests. J Exp Psychol Gen.

[20] Brewer R, Happe F, Cook R, Bird G (2015). Commentary on “Autism, oxytocin and interoception”: alexithymia, not autism spectrum disorders, is the consequence of interoceptive failure. Neurosci Biobehav Rev.

[21] Brewer R, Cook R, Bird G (2016). Alexithymia: a general deficit of interoception. R Soc Open Sci.

[22] Bottema-Beutel K, Kapp SK, Lester JN, Sasson NJ, Hand BN (2020). Avoiding ableist language: suggestions for autism researchers. Autism Adulthood..

[23] Bury SM, Jellett R, Spoor JR, Hedley D (2020). “It defines who I am” or “It’s something I have”: What language do autistic Australian adults on the autism spectrum prefer?. J Autism Dev Disord..

[24] Kenny L, Hattersley C, Molins B, Buckley C, Povey C, Pellicano E (2016). Which terms should be used to describe autism?. Perspect UK Autism Commun Autism.

[25] Kinnaird E, Stewart C, Tchanturia K (2019). Investigating alexithymia in autism: a systematic review and meta-analysis. Eur psychiatr.

[26] Improving the Measurement of Alexithymia in Autistic Adults: A Psychometric Investigation and Refinement of the Twenty-item Toronto Alexithymia Scale. 2021 Jan 27; Available from: https://www.researchsquare.com/article/rs-96364/v2. 10.1186/s13229-021-00427-9 PMC7971146 33653400

[27] Cai RY, Richdale AL, Uljarević M, Dissanayake C, Samson AC (2018). Emotion regulation in autism spectrum disorder: where we are and where we need to go. Autism Res.

[28] Garfinkel SN, Seth AK, Barrett AB, Suzuki K, Critchley HD (2015). Knowing your own heart: distinguishing interoceptive accuracy from interoceptive awareness. Biol Psychol.

[29] Brener J, Ring C (2016). Towards a psychophysics of interoceptive processes: the measurement of heartbeat detection. Phil Trans R Soc B.

[30] Fittipaldi S, Abrevaya S, de la Fuente A, Pascariello GO, Hesse E, Birba A (2020). A multidimensional and multi-feature framework for cardiac interoception. Neuroimage.

[31] Schandry R (1981). Heart beat perception and emotional experience. Psychophysiology.

[32] Smith AR, Dodd DR, Ortiz S, Forrest LN, Witte TK (2020). Interoceptive deficits differentiate suicide groups and associate with self-injurious thoughts and behaviors in a military sample. Suicide Life-Threaten Behav.

[33] Noel J-P, Lytle M, Cascio C, Wallace MT (2018). Disrupted integration of exteroceptive and interoceptive signaling in autism spectrum disorder. Autism Res.

[34] Fiene L, Brownlow C (2015). Investigating interoception and body awareness in adults with and without autism spectrum disorder. Autism Res.

[35] Palser ER, Fotopoulou A, Pellicano E, Kilner JM (2018). The link between interoceptive processing and anxiety in children diagnosed with autism spectrum disorder: extending adult findings into a developmental sample. Biol Psychol.

[36] Schauder KB, Mash LE, Bryant LK, Cascio CJ (2015). Interoceptive ability and body awareness in autism spectrum disorder. J Exp Child Psychol.

[37] Mash LE, Schauder KB, Cochran C, Park S, Cascio CJ (2017). Associations between interoceptive cognition and age in autism spectrum disorder and typical development. J Cogn Educ Psychol.

[38] Fiene L, Ireland MJ, Brownlow C (2018). The Interoception Sensory Questionnaire (ISQ): a scale to measure interoceptive challenges in adults. J Autism Dev Disord.

[39] Fujino H (2019). Further validation of the Japanese version of the multidimensional assessment of interoceptive awareness. BMC Res Notes.

[40] Mehling WE, Price C, Daubenmier JJ, Acree M, Bartmess E, Stewart A (2012). The multidimensional assessment of interoceptive awareness (MAIA). PLoS ONE.

[41] Rodriguez A, Reise SP, Haviland MG (2016). Applying bifactor statistical indices in the evaluation of psychological measures. J Pers Assess.

[42] Edwards MC, Houts CR, Cai L (2018). A diagnostic procedure to detect departures from local independence in item response theory models. Psychol Methods.

[43] Green SB, Hershberger SL (2000). Correlated errors in true score models and their effect on coefficient alpha. Struct Equ Model.

[44] Raykov T (2001). Estimation of congeneric scale reliability using covariance structure analysis with nonlinear constraints. Br J Math Stat Psychol.

[45] Yong AG, Pearce S (2013). A beginner’s guide to factor analysis: focusing on exploratory factor analysis. TQMP.

[46] Feliciano P, Daniels AM, Green Snyder L, Beaumont A, Camba A, Esler A (2018). SPARK: a US Cohort of 50,000 families to accelerate autism research. Neuron.

[47] Bagby RM, Parker JDA, Taylor GJ (1994). The twenty-item Toronto Alexithymia scale—I. Item selection and cross-validation of the factor structure. J Psychosomatic Res.

[48] John OP, Donahue EM, Kentle RL. Big Five inventory. American Psychological Association; 2012. Available from: 10.1037/t07550-000.

[49] R Core Team. R: A language and environment for statistical computing. Vienna, Austria; 2020. Available from: https://www.r-project.org/.

[50] Boyle GJ (1991). Does item homogeneity indicate internal consistency or item redundancy in psychometric scales?. Person Individ Differ.

[51] Li C-H (2016). Confirmatory factor analysis with ordinal data: comparing robust maximum likelihood and diagonally weighted least squares. Behav Res.

[52] Rosseel Y (2012). lavaan: an R package for structural equation modeling. J Stat Softw..

[53] Bentler PM (1990). Comparative fit indexes in structural models. Psychol Bull.

[54] Tucker LR, Lewis C (1973). A reliability coefficient for maximum likelihood factor analysis. Psychometrika.

[55] Steiger JH (1990). Structural model evaluation and modification: an interval estimation approach. Multivar Behav Res.

[56] Hu L, Bentler PM (1999). Cutoff criteria for fit indexes in covariance structure analysis: conventional criteria versus new alternatives. Struct Equ Model.

[57] Maydeu-Olivares A (2017). Assessing the size of model misfit in structural equation models. Psychometrika.

[58] DiStefano C, Liu J, Jiang N, Shi D (2018). Examination of the weighted root mean square residual: evidence for trustworthiness?. Struct Equ Model.

[59] Yu C-Y. Evaluating cutoff criteria of model fit indices for latent variable models with binary and continuous outcomes: University of California Los Angeles; 2002. Available from: https://www.statmodel.com/download/Yudissertation.pdf.

[60] Savalei V (2020). Improving fit indices in structural equation modeling with categorical data. Multivar Behav Res..

[61] Shi D, Maydeu-Olivares A, Rosseel Y (2020). Assessing fit in ordinal factor analysis models: SRMR vs. RMSEA. Struct Equ Model Multidiscip J.

[62] Marsh HW, Hau K-T, Wen Z (2004). In search of golden rules: Comment on hypothesis-testing approaches to setting cutoff balues for fit indexes and dangers in overgeneralizing Hu and Bentler’s (1999) findings. Struct Equ Model.

[63] McNeish D, An J, Hancock GR (2018). The thorny relation between measurement quality and fit index cutoffs in latent variable models. J Pers Assess.

[64] Tomarken AJ, Waller NG (2003). Potential problems with “well fitting” models. J Abnorm Psychol.

[65] Saris WE, Satorra A, van der Veld WM (2009). Testing structural equation models or detection of misspecifications?. Struct Equ Model.

[66] McDonald RP (1999). Test theory A unified treatment.

[67] Green SB, Yang Y (2009). Reliability of summed item scores using structural equation modeling: an alternative to coefficient alpha. Psychometrika.

[68] Kelley K, Pornprasertmanit S (2016). Confidence intervals for population reliability coefficients: evaluation of methods, recommendations, and software for composite measures. Psychol Methods.

[69] Samejima F (1969). Estimation of latent ability using a response pattern of graded scores. Psychometrika Monogr Suppl.

[70] Bock RD, Aitkin M (1981). Marginal maximum likelihood estimation of item parameters: application of an EM algorithm. Psychometrika.

[71] Chalmers RP. mirt : A multidimensional item response theory package for the *R* environment. J Stat Soft. 2012;48(6). Available from: http://www.jstatsoft.org/v48/i06/.

[72] Cai L, Monroe S. A new statistic for evaluating item response theory models for ordinal data. Los Angeles, CA: University of California, National Center for Research on Evaluation, Standards, and Student Testing (CRESST); 2014 p. 1–28. Report No.: CRESST Report 839. Available from: https://eric.ed.gov/?id=ED555726.

[73] Monroe S, Cai L (2015). Evaluating structural equation models for categorical outcomes: a new test statistic and a practical challenge of interpretation. Multivar Behav Res.

[74] Maydeu-Olivares A, Joe H (2014). Assessing approximate fit in categorical data analysis. Multivar Behav Res.

[75] Chen W-H, Thissen D (1997). Local dependence indexes for item pairs using item response theory. J Educ Behav Stat.

[76] Toland MD (2014). Practical guide to conducting an item response theory analysis. J Early Adolesc.

[77] Cao M, Tay L, Liu Y (2017). A Monte Carlo study of an iterative wald test procedure for DIF analysis. Educ Psychol Meas.

[78] Williams ZJ. irt_extra: Additional functions to supplement the mirt R package. Nashville, TN; 2020 . Available from: http://rgdoi.net/10.13140/RG.2.2.10226.04803/1.

[79] Benjamini Y, Hochberg Y (1995). Controlling the false discovery rate: a practical and powerful approach to multiple testing. J Roy Stat Soc Ser B (Methodol).

[80] Savalei V, Reise SP (2019). Don’t forget the model in your model-based reliability coefficients: a reply to McNeish (2018). Collabra Psychol.

[81] Reise SP, Moore TM, Sabb FW, Brown AK, London ED (2013). The barratt impulsiveness scale–11: reassessment of its structure in a community sample. Psychol Assess.

[82] McNeish D, Wolf MG (2020). Thinking twice about sum scores. Behav Res..

[83] Simms LJ, Zelazny K, Williams TF, Bernstein L (2019). Does the number of response options matter? Psychometric perspectives using personality questionnaire data. Psychol Assess.

[84] Flake JK, Fried EI. Measurement schmeasurement: Questionable measurement practices and how to avoid them. Advances in Methods and Practices in Psychological Science. 2020 Dec;3(4):456-65.

[85] Loomes R, Hull L, Mandy WPL (2017). What Is the male-to-female ratio in autism spectrum disorder? A systematic review and meta-analysis. J Am Acad Child Adolesc Psychiatry.

[86] Bitsika V, Sharpley CF, Mills R (2018). Sex differences in sensory features between boys and girls with autism spectrum disorder. Res Autism Spectrum Disord.

[87] Ross LA, Del Bene VA, Molholm S, Frey H-P, Foxe JJ. Sex differences in multisensory speech processing in both typically developing children and those on the autism spectrum. Front Neurosci. 2015;9. Available from: https://www.frontiersin.org/articles/10.3389/fnins.2015.00185/full. 10.3389/fnins.2015.00185 PMC4445312 26074757

[88] Daniels AM, Rosenberg RE, Anderson C, Law JK, Marvin AR, Law PA (2012). Verification of parent-report of child autism spectrum disorder diagnosis to a web-based autism registry. J Autism Dev Disord.

