# Supplementary material for: Psychometric validation and refinement of the Interoception Sensory Questionnaire (ISQ) in adolescents and adults on the autism spectrum
Source: Mol Autism. 2021 Jun 7;12:42. doi: 10.1186/s13229-021-00440-y (PMC8185943; doi:10.1186/s13229-021-00440-y)
Supplement: Supplementary file 1 — Additional file 1. Supplemental figures and tables. [file 13229_2021_440_MOESM1_ESM.docx]

**Supplementary Materials**

**Supplemental Table S1**

*All Misspecified Item Pairs and Those with High Power*

| Item pair | | Modification Index Value | EPC [95% CI] |
| --- | --- | --- | --- |
| 19 | 20 | 138.030 | 0.25 [0.215, 0.285] |
| 5 | 13 | 96.386 | 0.164 [0.136, 0.191] |
| 3 | 11 | 74.071 | 0.155 [0.126, 0.185] |
| 6 | 14 | 45.057 | 0.152 [0.115, 0.189] |
| 3 | 8 | 41.664 | 0.11 [0.082, 0.137] |
| 8 | 11 | 29.943 | 0.122 [0.086, 0.159] |
| 2 | 3 | 28.909 | 0.157 [0.109, 0.205] |
| 7 | 19 | 27.466 | 0.116 [0.08, 0.152] |
| 15 | 16 | 26.904 | 0.159 [0.109, 0.21] |
| 6 | 19 | 18.371 | 0.137 [0.084, 0.189] |
| 7 | 20 | 18.245 | -0.156 [-0.216, -0.096] |
| 6 | 11 | 13.505 | -0.11 [-0.16, -0.061] |
| 14 | 19 | 12.869 | -0.11 [-0.16, -0.06] |
| 3 | 6 | 12.562 | -0.12 [-0.176, -0.064] |
| 3 | 18 | 12.226 | -0.118 [-0.174, -0.063] |
| 3 | 14 | 11.932 | -0.113 [-0.167, -0.059] |
| 6 | 8 | 10.193 | -0.111 [-0.168, -0.054] |
| 8 | 14 | 10.156 | -0.112 [-0.169, -0.054] |
| 16 | 19 | 9.971 | -0.106 [-0.162, -0.051] |
| 11 | 20 | 9.809 | -0.143 [-0.218, -0.068] |
| 6 | 7 | 8.894 | -0.116 [-0.18, -0.052] |
| 8 | 18 | 8.851 | -0.109 [-0.168, -0.049] |
| 13 | 20 | 8.837 | -0.103 [-0.161, -0.046] |
| 1 | 11 | 8.021 | -0.11 [-0.173, -0.046] |
| 7 | 14 | 7.629 | -0.103 [-0.165, -0.042] |
| 5 | 20 | 7.206 | -0.109 [-0.175, -0.042] |
| 7 | 9 | 6.459 | -0.103 [-0.169, -0.036] |
| 2 | 7 | 5.953 | 0.095 [0.031, 0.159] |
| 1 | 19 | 5.595 | -0.088 [-0.149, -0.027] |
| 4 | 7 | 5.446 | -0.091 [-0.155, -0.027] |
| 1 | 13 | 5.143 | -0.084 [-0.145, -0.023] |
| 4 | 18 | 4.703 | -0.082 [-0.144, -0.02] |
| 7 | 11 | 4.473 | -0.09 [-0.16, -0.02] |

**Supplemental Table S2**

*Interoceptive Sensory Questionnaire Short Form (ISQ-8)*

|  | **Not true at all of me** |  |  |  | **Very true of me** |
| --- | --- | --- | --- | --- | --- |
| 1. I have difficulty making sense of my body’s signals unless they are very strong |  |  |  |  |  |
| 2. I have difficulty feeling my bodily need for food |  |  |  |  |  |
| 3. I’m not sure how my body feels when it’s a hot day |  |  |  |  |  |
| 4. I find it difficult to describe feelings like hunger, thirst, hot or cold |  |  |  |  |  |
| 5. There are times when I am only aware of changes in my body because of the reactions of other people |  |  |  |  |  |
| 6. I find it difficult to identify some of the signals that my body is telling me (e.g., If I’m about to faint or I’ve overexerted myself) |  |  |  |  |  |
| 7. I have difficulty locating injury in my body |  |  |  |  |  |
| 8. Even when I know that I am physically uncomfortable, I do not act to change my situation |  |  |  |  |  |

**Supplemental Table S3**

*Differential Item Functioning (DIF) using Iterative Wald Test Procedure for Participant Age, Sex, Gender, and Household Income*

***Age***

| **Item #** | **χ^2^** | **df** | ***p*-value^a^** |
| --- | --- | --- | --- |
| 1 | 2.405 | 7 | 0.934 |
| 3 | 6.962 | 7 | 0.934 |
| 4 | 7.237 | 7 | 0.934 |
| 5 | 2.469 | 7 | 0.934 |
| 9 | 4.29 | 7 | 0.934 |
| 12 | 2.732 | 7 | 0.934 |
| 15 | 3.394 | 7 | 0.934 |
| 20 | 8.051 | 7 | 0.934 |

|  |  |  |  |
| --- | --- | --- | --- |
| **Sex**   \| **Item #** \| **χ^2^** \| **df** \| ***p*-value^a^** \| \| --- \| --- \| --- \| --- \| \| 1 \| 5.554 \| 7 \| 0.576 \| \| 3 \| 6.923 \| 7 \| 0.576 \| \| 4 \| 6.31 \| 7 \| 0.576 \| \| 5 \| 11.652 \| 7 \| 0.576 \| \| 9 \| 2.897 \| 7 \| 0.894 \| \| 12 \| 7.565 \| 7 \| 0.576 \| \| 15 \| 6.627 \| 7 \| 0.576 \| \| 20 \| 8.349 \| 7 \| 0.576 \| |  |  |  |
| ^a^ Benjamini & Hochberg correction used |  |  |  |

**Gender**

| **Item #** | **χ^2^** | **df** | ***p*-value^a^** |
| --- | --- | --- | --- |
| 1 | 9.046 | 7 | 0.707 |
| 3 | 5.483 | 7 | 0.707 |
| 4 | 5.341 | 7 | 0.707 |
| 5 | 7.628 | 7 | 0.707 |
| 9 | 2.93 | 7 | 0.891 |
| 12 | 9.087 | 7 | 0.707 |
| 15 | 5.848 | 7 | 0.707 |
| 20 | 8.211 | 7 | 0.707 |

**Household Income**

| **Item #** | **χ^2^** | **df** | ***p*-value^a^** |
| --- | --- | --- | --- |
| 1 | 13.501 | 7 | 0.191 |
| 3 | 4.554 | 7 | 0.992 |
| 4 | 14.97 | 7 | 0.191 |
| 5 | 3.001 | 7 | 0.992 |
| 9 | 13.021 | 7 | 0.191 |
| 12 | 5.36 | 7 | 0.992 |
| 15 | 3.025 | 7 | 0.992 |
| 20 | 1.142 | 7 | 0.992 |

***Age***

| **Item #** | **χ^2^** | **df** | ***p*-value^a^** |
| --- | --- | --- | --- |
| 1 | 8.921 | 5 | 0.299 |
| 3 | 10.826 | 5 | 0.220 |
| 4 | 14.524 | 5 | 0.101 |
| 5 | 4.455 | 5 | 0.777 |
| 9 | 2.890 | 5 | 0.904 |
| 12 | 4.633 | 5 | 0.777 |
| 15 | 2.404 | 5 | 0.904 |
| 20 | 1.357 | 5 | 0.929 |

|  |  |  |  |
| --- | --- | --- | --- |
| **Sex**   \| **Item #** \| **χ^2^** \| **df** \| ***p*-value^a^** \| \| --- \| --- \| --- \| --- \| \| 1 \| 1.959 \| 5 \| 0.94 \| \| 3 \| 1.245 \| 5 \| 0.94 \| \| 4 \| 2.122 \| 5 \| 0.94 \| \| 5 \| 2.202 \| 5 \| 0.94 \| \| 9 \| 3.081 \| 5 \| 0.94 \| \| 12 \| 3.657 \| 5 \| 0.94 \| \| 15 \| 4.922 \| 5 \| 0.94 \| \| 20 \| 2.442 \| 5 \| 0.94 \| |  |  |  |
| ^a^ Benjamini & Hochberg correction used |  |  |  |

**Gender**

| **Item #** | **χ^2^** | **df** | ***p*-value^a^** |
| --- | --- | --- | --- |
| 1 | 1.229 | 5 | 0.942 |
| 3 | 1.237 | 5 | 0.942 |
| 4 | 3.096 | 5 | 0.942 |
| 5 | 2.742 | 5 | 0.942 |
| 9 | 1.744 | 5 | 0.942 |
| 12 | 5.227 | 5 | 0.942 |
| 15 | 4.225 | 5 | 0.942 |
| 20 | 2.833 | 5 | 0.942 |

**Household Income**

| **Item #** | **χ^2^** | **df** | ***p*-value^a^** |
| --- | --- | --- | --- |
| 1 | 4.594 | 5 | 0.759 |
| 3 | 4.608 | 5 | 0.759 |
| 4 | 7.557 | 5 | 0.759 |
| 5 | 3.862 | 5 | 0.759 |
| 9 | 3.956 | 5 | 0.759 |
| 12 | 3.911 | 5 | 0.759 |
| 15 | 2.232 | 5 | 0.759 |
| 20 | 1.316 | 5 | 0.759 |

**Supplemental Figure S4**

*Trace Lines for ISQ-20 Item 3 Before and After Collapsing from 7 to 5 Categories*

**ISQ-20 ISQ-8**
